# *Bacillus coagulans*–Pectin Synbiotic Modulates Gut Microbiota Composition and Attenuates Ethanol-Induced Alcoholic Liver Disease in Mice

**DOI:** 10.3390/microorganisms13091986

**Published:** 2025-08-26

**Authors:** Zhenzhen Liu, Tong Liu, Yurong Fan, Ziyang Jiang

**Affiliations:** 1 Anti-Infective Agent Creation Engineering Research Centre of Sichuan Province, Antibiotics Research and Re-Evaluation Key Laboratory of Sichuan Province, Sichuan Industrial Institute of Antibiotics, School of Pharmacy, Chengdu University, Chengdu 610106, China; 2 State Key Laboratory of Agricultural Microbiology, College of Life Science and Technology, Huazhong Agricultural University, Wuhan 430070, China

**Keywords:** *Bacillus coagulans*, pectin, alcoholic liver diseases, microbiota, intestinal barrier

## Abstract

Alcohol abuse and alcoholic liver diseases (ALD) are globally prevalent, with alcohol-induced gut microbiota dysbiosis playing a key role in ALD pathogenesis. Synbiotic (combinations of probiotics and prebiotics) are recognized as effective in reducing inflammation in ALD. *Bacillus coagulans*, a probiotic with favorable industrial and functional traits (e.g., sporulation, lactic acid production), shows potential in treating intestinal diseases. Here, we investigated the effects of *B. coagulans*, alone or combined with pectin, on ethanol-induced ALD in mice. Synbiotic supplementation (*B. coagulans* + pectin) more significantly alleviated ethanol-induced ALD severity than *B. coagulans* or pectin alone. Relative to the ethanol group, synbiotic treatment significantly reduced hepatic inflammatory injury and lipid accumulation, downregulated proinflammatory factors (TNF-α, IL-1β, myeloperoxidase [MPO]), and upregulated tight junction proteins and mucins—enhancing intestinal barrier function. Moreover, these supplements modulated gut microbiota composition and enhanced short-chain fatty acids (SCFAs) production by increasing the abundance of beneficial SCFA-producing bacteria (*Muribaculaceae*, *Akkermansia*). In summary, changes in tight junction proteins, cytokines and hepatic injury markers indicate that the synbiotic alleviated overall inflammation in the experimental ALD model and exerted a greater therapeutic effect than *B. coagulans* or pectin alone.

## 1. Introduction

Alcoholic liver disease (ALD), a consequence of chronic alcohol consumption, is a leading contributor to liver-related morbidity and mortality globally [1]. Notably, alcohol alone accounted for approximately 2.6 million global deaths in 2019, a statistic that underscores both the severity of this public health crisis and the urgent need for effective preventive and therapeutic interventions [2]. As the most prevalent form of chronic liver disease globally, ALD progresses through distinct stages: it begins with alcoholic fatty liver (AFL), advances to alcoholic steatohepatitis (ASH) which is defined by hepatic inflammation, and may ultimately progress to fibrosis, cirrhosis, or even hepatocellular cancer (HCC) in persistent cases. Additionally, severe ASH (with or without co-existing cirrhosis) can precipitate alcoholic hepatitis an acute ALD manifestation associated with liver failure and elevated mortality [3,4]. Given the rising global incidence of ALD in recent years, identifying effective therapeutic strategies has become increasingly critical.

Mounting evidence indicates that ethanol-induced oxidative stress and innate immunological responses, which contribute to hepatocyte injury, play pivotal roles in ALD pathogenesis [5,6,7]. Currently, three potential therapeutic approaches for ALD are under consideration: (i) strategies to mitigate hepatic injury by reducing oxidative stress, minimizing hepatocyte death, and enhancing hepatic repair; (ii) anti-inflammatory agents, such as the traditional drug prednisolone; and (iii) gut-targeted interventions, including probiotic administration and fecal microbiota transplantation [1]. Currently, only a few drugs such as acamprosate, disulfiram, and naltrexone have been approved by the Food and Drug Administration (FDA) for the treatment of ALD patients [8]. However, long-term use of these drugs often leads to increased drug resistance and elevated infection risks, so it is imperative to explore new drugs capable of preventing and treating ALD and its related diseases.

Probiotics are “rigorously selected live microorganisms that, when administered in sufficient quantities, confer health benefits to the host” [9]. Their therapeutic mechanisms include immune modulation, gut microbiota interaction, barrier enhancement, mediation of host interactions via surface structures, and organic acids production [10]. While clinical guidelines exist for probiotics use in pediatric [11], with evidence supporting their systemic effects in reducing upper respiratory tract infection incidence and duration [12], formal recommendations for adults are currently lacking [13]. Post-probiotic supplementation, a healthy gut microbiota is crucial for ALD treatment [14], influencing immune system, intestinal barrier, and intestinal structure [15]. Prebiotics, defined as “selectively fermented substrates that confer specific health benefits via host–microbe interactions” [16], offer benefits including pathogen defense, immune modulation, enhanced mineral absorption, bowel regulation, metabolic effects, and satiety promotion [10]. Synbiotic therapies (probiotics and prebiotics) show promise: probiotics introduce commensal bacteria that compete with pathogens for nutrients/adhesion sites or produce inhibitory metabolites, while prebiotics selectively promote beneficial microbial growth [17]. However, the evidence base for prebiotic lags behind that for probiotics. Although significant effects are documented in infant formula (e.g., GOS/fructan mixtures reducing respiratory infections to breastfed levels, GOS reducing diarrhea incidence) [18,19], evidence for prebiotics reducing infections in adults is limited. Research primarily focuses on functional foods, necessitating further clinical evidence accumulation.

As functional foods, probiotics and prebiotics restore alcohol-induced intestinal barrier dysfunction and mitigate alterations to gut microbiota [14,20,21]. *Bacillus coagulans*, notable for its endospore formation [22], exhibits exceptional environmental tolerance. Recognized as safe with a history of food use, it has been granted GRAS (Generally Recognized As Safe) status from the USFDA and approval from China’s National Health Commission. Its stability facilitates widespread application in pharmaceutical, food, chemical and other industries. Recent studies demonstrate *B. coagulans* efficacy against intestinal diseases, including acute diarrhea, irritable bowel syndrome, antibiotic-associated diarrhea, constipation, and colitis by modulation of microbiota composition, host immunity, and metabolism [23,24]. Furthermore, it acts as an immunomodulator, suppressing LPS-induced inflammation via regulation of proinflammatory and anti-inflammatory cytokines [25,26,27]. Our previous studies confirmed its ability to ameliorate ALD in mice by modulating intestinal microbiota [28], indicating its role in mitigating inflammation-driven tissue damage and enhancing host immune defense. Pectin, a prebiotic, improves intestinal barrier integrity and alters gut microbiota to prevent ALD in mice [29,30,31]. This complex heteropolysaccharide primarily consists of linear galacturonic acid (GalA) chains linked to branched rhamnogalacturonan regions [32,33]. Pectin structure significantly influences nutrient absorption, gut microbial composition, and metabolite profiles [32]. Synergistic effects between probiotics and prebiotics enhance probiotic efficacy [26,34], establishing synbiotic (probiotic-prebiotic) combinations as a promising novel therapeutic strategy for ALD.

Building on our prior findings that *B. coagulans* ameliorates ALD in mice [28], this study employed a probiotic-prebiotic (synbiotic) combination strategy to investigate its efficacy against ethanol-induced ALD. We assessed the impact on host immune, gut microbiota composition, colonic barrier integrity, and hepatic histopathology, measuring key indicators including cytokines (TNF-α, IL-1β), myeloperoxidase (MPO), tight junction proteins, and mucin proteins. The results elucidate the biological mechanism underlying the improved ALD prognosis with *B. coagulans*–pectin co-administration, providing novel insights for developing ALD therapeutics.

## 2. Materials and Methods

### 2.1. Animals

Fifty 8-week-old C57BL/6 mice (average weight: 25 g) were obtained from the Center for Experimental Animals, Huazhong Agricultural University, Wuhan, China. The mice were housed under controlled conditions at 25 °C with a 12 h day/night light cycle. Body weights were measured daily, including during the first 7-day acclimation period. All experimental procedures involving animals were approved by the Animal Ethics Committee of Laboratory Animal Center, Huazhong Agriculture University (Ethics Approval Number: HZAUMO-2022-0138, approval date: 1 September 2022).

### 2.2. Study Design and Treatments

Bertola et al. [35] established an ALD model in mice using a Lieber–DeCarli ethanol-containing diet, combining 10 days of ad libitum feeding with a Lieber–DeCarli ethanol liquid diet and a single binge ethanol gavage to induce ALD. This chronic-binge feeding protocol synergistically induces liver injury, inflammatory response, and steatosis, effectively mimicking the pathological characteristics of human acute-on-chronic alcoholic liver injury. In the present study, all mice underwent 5 consecutive days of Lieber–DeCarli pair-feeding to acclimatize to liquid diet and tube feeding. They were then randomly divided into 5 groups (10 mice per group, 5 mice per cage): (1) healthy control group; (2) ethanol-treated ALD model group; (3) ethanol-treated group supplemented with *B. coagulans* FCYS01 (*B.co* group); (4) ethanol-treated group supplemented with pectin (pectin group); (5) ethanol-treated group supplemented with both *B. coagulans* and pectin (synbiotic group). Mice in the ALD model group and treatment groups were fed a Lieber–DeCarli ethanol diet (5% ethanol), with caloric intake matched to that of healthy control group mice. Starting from day 9, mice in the *B.co* group were gavaged with *B. coagulans* (1.0 × 10^9^ CFU) every 2 days as the treatment; the pectin group received a Lieber–DeCarli diet containing 1% pectin; the synbiotic group was administered both a 1% pectin-containing Lieber–DeCarli diet and gavage of *B. coagulans* (1.0 × 10^9^ CFU) every 2 days. The control group was gavaged with an equal volume of PBS. On day 16, the ALD model and treatment groups were gavaged with ethanol (5 g/kg body weight), while the healthy control group was received intragastric administration of isocaloric maltodextrin. Nine hours after gavage, the mice were euthanized, and samples were collected.

Plasma samples were collected and subjected to centrifugation at 10,000× *g*, 10 min, 4 °C. Supernatants were then stored at −80 °C for ELISA and biochemical testing. Liver and colon tissues were harvested immediately, washed with PBS, and frozen at −80 °C for subsequent analyses.

### 2.3. Serum and Liver Biochemical Assays Combined with ELISA Analyses

Hepatic interleukin-1β (IL-1β, Jiangsu Meimian Industrial Co., Ltd., Yancheng, China, MM-0040M1), myeloperoxidase (MPO, Jiangsu Meimian Industrial Co., Ltd., MM-0338M1), tumor necrosis factor alpha (TNF-α, Jiangsu Meimian Industrial Co., Ltd., MM-0132M1) and serum alanine transaminase (ALT, Nanjing Jiancheng Co., Ltd., Nanjing, China, C009-2-1), aspartate transaminase (AST, Nanjing Jiancheng Co., Ltd., C010-2-1), lipopolysaccharide (LPS, Jiangsu Meimian Industrial Co., Ltd., MM-0634M1) levels were measured using ELISA kits. Biochemical kits were utilized to ascertain the amounts of hepatic triglyceride (TG, Nanjing Jiancheng Co., Ltd., A110-1-1).

### 2.4. Real-Time Quantitative PCR (RT-qPCR)

Total RNA was extracted from tissue samples using the RNAeasy™ Animal RNA Isolation Kit. cDNA synthesis was performed by PCR using HiScript^®^ II Q RT SuperMix for qPCR (Vazyme, Nanjing, China) with the following program: 42 °C for 2 min, 50 °C for 15 min, and 85 °C for 5 s. Real-time quantitative PCR was run on a QuantStudio3 (Thermo Fisher Scientific, Waltham, PA, USA) with the Taq Pro Universal SYBR qPCR Master Mix (Vazyme, Nanjing, China). The PCR protocol was as follows: 95 °C for 30 s (initial denaturation), followed by 40 cycles of 95 °C for 5 s, 60 °C for 10 s, and 72 °C for 20 s. Gene expression levels were calculated using the comparative Ct method (2^−ΔΔCt^). Each cDNA sample was analyzed in duplicate to ensure quantitative accuracy of RNA amplification. Table S1 contains a summary of the primers that were utilized.

### 2.5. Growth Curves Test

1% overnight-cultured *B. coagulans* was added to YPD liquid medium (1% Yeast Extract, 2% Peptone and 2% Dextrose (glucose)), medium containing 2% pectin as a carbon source (YP + pectin) and medium without carbon source (YP). The media was then incubated at 37 °C, with the OD_600_ value being measured every four hours.

### 2.6. Staining Procedures

According to guidelines, liver samples underwent formalin-fixed and sectioning for hematoxylin-eosin (H&E) staining [36], while frozen liver sections were stained with Oil red O [36]. A Nikon Eclipse 80i microscope (Nikon, Kobe, Japan) was used to scan every section.

### 2.7. Metagenome Sequencing and Bioinformatics

At the conclusion of the experiment, fecal DNA samples were collected from the cecum in each group for subsequent metagenomic sequencing and analysis. After being extracted, DNA was used to sequence data using an Illumina HiSeq 2500 instrument (San Diego, CA, USA). Trimmomatic software 0.39 was used to apply quality control settings to the data received during sequencing, using the settings: LEADING:3 TRAILING:3 SLIDINGWINDOW:5:20 MINLEN:50. Subsequent quality control of the Clean Data for DE novo splice (K—killing choose K—min 35 K—Max 95, K—step 20) using MEGAHIT (https://github.com/voutcn/megahit, accessed on 20 November 2022). To capture low-abundance microbial taxa in the samples, unassembled reads from individual samples were pooled for co-assembly. The sequence fragments without N, known as Scaftig, were obtained by breaking the built scaffolds from the N-junction. For further examination, snippets that had spliced lengths longer than 500 bp were screened. Open reading frames (ORFs) were predicted using MetaGeneMark from Scaftigs of single sample and hybrid assembly; the number and length of the projected ORFs were assessed. Unigenes were aligned to the NCBI NR database using BLASTP (Version 2.2.31+; e-value ≤ 0.0001). Taxonomic annotations were assigned using the LCA (Lowest Common Ancestor) algorithm implemented in MEGAN software 7, based on the first branching node. Microbial composition and relative abundance across samples were further analyzed using read-based Metaphlan software 2. The metagenomic sequencing datasets have been submitted to the NCBI Sequence Read Archive (SRA) under accession number: PRJNA1044840.

### 2.8. Quantification of SCFAs

We refer to the reported method for the rapid determination of SCFAs in feces via acidified water extraction coupled with direct injection gas chromatography [37]. SCFAs, including acetate, propionate, and butyrate in fecal samples were quantified with gas chromatography. Briefly, 50 mg of fecal samples were weighed, dissolved, homogenized, and then centrifuged at 3000× *g* for 5 min. The supernatant was adjusts pH to 2 to 3 with HCl, and then centrifuged at 12,000× *g* for 10 min, the last filtered through a 0.22 μm sterile membrane, kept in a 2 mL screw-cap vial, and then subjected for SCFAs analysis with an gas chromatography system (Agilent Technologies 8860-5977C, Santa Clara, CA, USA).

### 2.9. Statistical Analysis

We utilized GraphPad Prism 8 and SPSS Statistics 22 for data analysis. When appropriate, an ordinary one-way ANOVA was employed, followed by Tukey’s back comparison test. At *p* < 0.05, statistical significance was taken into consideration.

## 3. Results

### 3.1. B. coagulans, Pectin and the Synbiotic Alleviated Ethanol-Induced Liver Histological Damage in Mice

**Figure 1:**
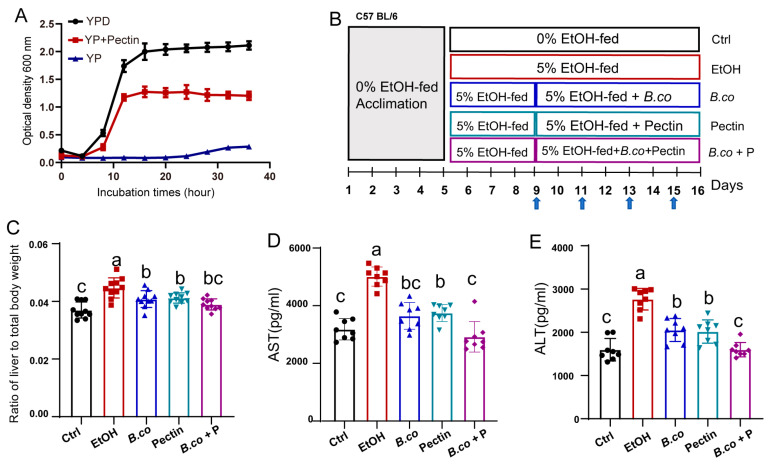
Treatment effect of *B. coagulans*, pectin and the synbiotic on ethanol-induced ALD mice. (**A**) Growth curves of *B. coagulans* in YPD, medium with pectin as carbon source (YP + Pectin) and medium without carbon source (YP). (**B**) Schematic representation of the ethanol-induced ALD mouse model utilized in this study. Arrows denote intragastric administration of phosphate-buffered saline (PBS) or *B. coagulans* treatments. (**C**) Ratio of liver weight to body weight. (**D**) Serum AST level. (**E**) Serum ALT level. Data are presented as means ± SEM. Values with distinct superscript letters (a, b and c) indicate significant differences (*p* < 0.05) as determined by one-way ANOVA followed by Tukey’s test. Ctrl: healthy control group; EtOH: ethanol-induced group; *B. co*: supplementation of *B. coagulans* group; Pectin: supplementation of pectin group; *B. co* + P: supplementation of *B. coagulans* and pectin group.

*B. coagulans* utilized pectin as a carbon source for growth (YP + Pectin), although less efficiently than glucose (YPD) (Figure 1A). Based on this, we investigated the therapeutic potential of *B. coagulans* and pectin in an ethanol-induced ALD mouse model. Mice received a Lieber–DeCarli liquid diet: healthy control group for 16 days, while ethanol treatment groups started receiving a 5% ethanol on day 5. From day 9, treatment groups received *B. coagulans* by gavage every two days and/or a 1% pectin-supplemented diet (Figure 1B). All treatment groups significantly reduced the ethanol-induced liver-to-total body weight ratios (Figure 1C), a sensitive indicator of liver volume, and consistent trends were observed in serum levels of liver injury markers aspartate aminotransferase (AST) (Figure 1D) and alanine aminotransferase (ALT) (Figure 1E). Compared to the ethanol model group, supplementation with *B. coagulans* pectin, or their synbiotic combination significantly ameliorated hepatic lesions. The synbiotic treatment group exhibited the most pronounced improvement, with values approaching those of the healthy control group (Figure 1).

**Figure 2:**
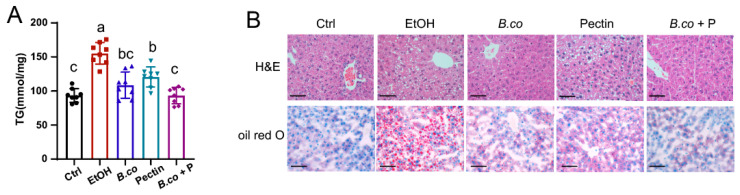
*B. coagulans*, pectin and the synbiotic reduced histological damage on ethanol-induced ALD mice. (**A**) Liver triglyceride concentration. (**B**) Histological assessment of steatosis in liver sections, with representative micrographs of H&E staining (**top**) and Oil Red O staining (**bottom**). Scale bars = 50 μm. Data are presented as means ± SEM. Values with distinct superscript letters (a, b and c) indicate significant differences (*p* < 0.05) as determined by one-way ANOVA followed by Tukey’s test. Ctrl: healthy control group; EtOH: ethanol-induced group; *B. co*: supplementation of *B. coagulans* group; Pectin: supplementation of pectin group; *B. co* + P: supplementation of *B. coagulans* and pectin group.

Hepatic triglyceride (TG) quantification and histopathological staining further assessed the effects of *B. coagulans*, pectin, and their synbiotic on liver histology in ethanol-induced mice. Ethanol intake significantly elevated hepatic TG levels, indicative of impaired lipid metabolism (Figure 2A). Treatments with *B. coagulans*, pectin, and their synbiotic significantly reduced this ethanol-induced TG accumulation (Figure 2A). Histologically (H&E and Oil Red O staining), ethanol exposure caused significant macrovesicular fat accumulation, hepatocyte ballooning, and cytoplasmic lipid vacuolization compared to healthy control group (Figure 2B). All treatments ameliorated these ethanol-induced liver lesions, restoring hepatic histology towards normalcy. Notably, the synbiotics intervention yielded the most substantial recovery, approaching healthy control histology (Figure 2B). These findings demonstrate that *B. coagulans*, pectin, and particularly their synbiotic combination effectively restore ethanol-induced liver injury.

### 3.2. B. coagulans, Pectin and Their Synbiotic Improved Immune Regulation in Ethanol-Induced Mice

**Figure 3:**
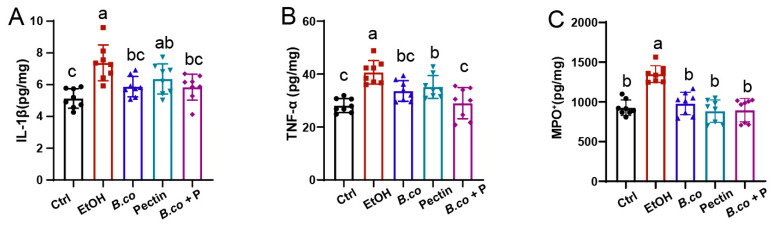
Regulatory effects of *B. coagulans*, pectin and their synbiotic on immune markers in ethanol-induced mice. ELISA was used to analyze the protein levels of hepatic cytokines, including (**A**) IL-1β, (**B**) TNF-α and (**C**) MPO. Data are presented as means ± SEM. Values with distinct superscript letters (a, b and c) indicate significant differences (*p* < 0.05) as determined by one-way ANOVA followed by Tukey’s test. Ctrl: healthy control group; EtOH: ethanol-induced group; *B. co*: supplementation of *B. coagulans* group; Pectin: supplementation of pectin group; *B. co* + P: supplementation of *B. coagulans* and pectin group.

To assess the impact on hepatic inflammation, we quantified proinflammatory cytokines TNF-α and IL-1β in liver tissues. Ethanol administration significantly increased IL-1β (Figure 3A) and TNF-α (Figure 3B) levels. Supplementation with *B. coagulans*, pectin, or synbiotic significantly attenuated these increases. *B. coagulans* and the synbiotic demonstrated greater efficacy than pectin alone in reducing IL-1β levels. Furthermore, the synbiotic induced a significantly greater reduction in TNF-α levels compared to either *B. coagulans* or pectin supplementation alone. Analysis of myeloperoxidase (MPO) content, an indicator of inflammatory cell infiltration, revealed that all three treatments significantly reduced ethanol-induced MPO elevation (Figure 3C). Collectively, these findings indicate that *B. coagulans*, pectin, and their synbiotic effectively mitigate ethanol-induced hepatic inflammation, with the synbiotic treatment exhibiting a superior therapeutic effect on ethanol-induced ALD in mice.

### 3.3. B. coagulans, Pectin and Their Synbiotic Formulation Ameliorated the Impaired Barrier Function in Ethanol-Induced Mice

**Figure 4:**
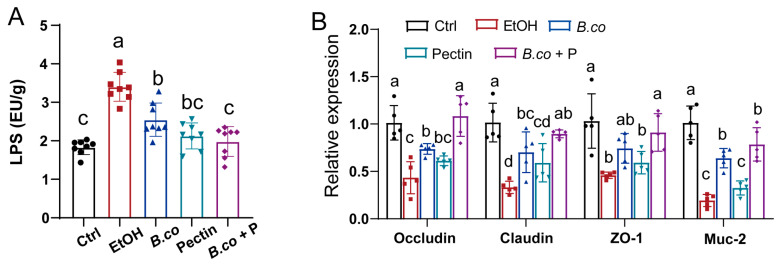
*B. coagulans*, pectin and their synbiotic formulation ameliorated the impaired barrier function in ethanol-induced mice. (**A**) Serum lipopolysaccharide levels. (**B**) The mRNA levels of tight junction proteins (Occludin, Claudin and ZO-1) and mucin protein Muc2 were detected by RT-qPCR analysis in colon. Data are presented as means ± SEM. Values with distinct superscript letters (a, b and c) indicate significant differences (*p* < 0.05) as determined by one-way ANOVA followed by Tukey’s test. Ctrl: healthy control group; EtOH: ethanol-induced group; *B. co*: supplementation of *B. coagulans* group; Pectin: supplementation of pectin group; *B. co* + P: supplementation of *B. coagulans* and pectin group.

Ethanol exposure attenuates the intestinal mucus layer and disrupts mucosal barrier function [20]. This dysfunction, closely linked to ALD progression, promotes translocation of microbial products (e.g., LPS) from the gut to the liver [38,39]. Alcohol-induced dysbiosis and increased intestinal permeability further facilitate this process [39]. To investigate protective mechanisms against ALD, serum LPS levels were measured. Ethanol intake significantly elevated serum LPS compared to healthy control group (Figure 4A), confirming intestinal barrier disruption and subsequent LPS leakage. Supplementation with *B. coagulans*, pectin, or synbiotic significantly reduced LPS levels. Notably, *B. coagulans* was less effective at decreasing LPS levels than pectin or the synbiotic (Figure 4A), suggesting these treatments help maintain intestinal barrier integrity in model of ethanol-induced ALD.

The impact on intestinal barrier integrity was further assessed via tight junction (TJ) protein expression (Occludin, Claudin, ZO-1). Ethanol significantly downregulated TJ protein mRNA expression levels compared to the healthy control group (Figure 4B). *B. coagulans* and their synbiotic supplementation significantly increased the expression of TJ protein, with the synbiotic showing the greatest efficacy; pectin group had minimal effect (Figure 4B). Similarly, ethanol markedly reduced expression of Muc2, the primary mucin constituent critical for the mucus layer (Figure 4B). Expression of Muc2 was significantly upregulated in the *B. coagulans* and synbiotic groups compared to the ethanol-induced group (Figure 4B), likely enhancing mucus production and barrier recovery. Overall, *B. coagulans* and the synbiotic enhance expression of TJ and mucin proteins, thereby improving intestinal integrity and barrier function in ethanol-induced damage mice. The synbiotic supplementation demonstrated superior efficacy in treating ethanol-induced ALD in mice.

### 3.4. B. coagulans, Pectin and Their Synbiotic Supplementation Regulated the Composition and SCFAs Production of Gut Microbiota

**Figure 5:**
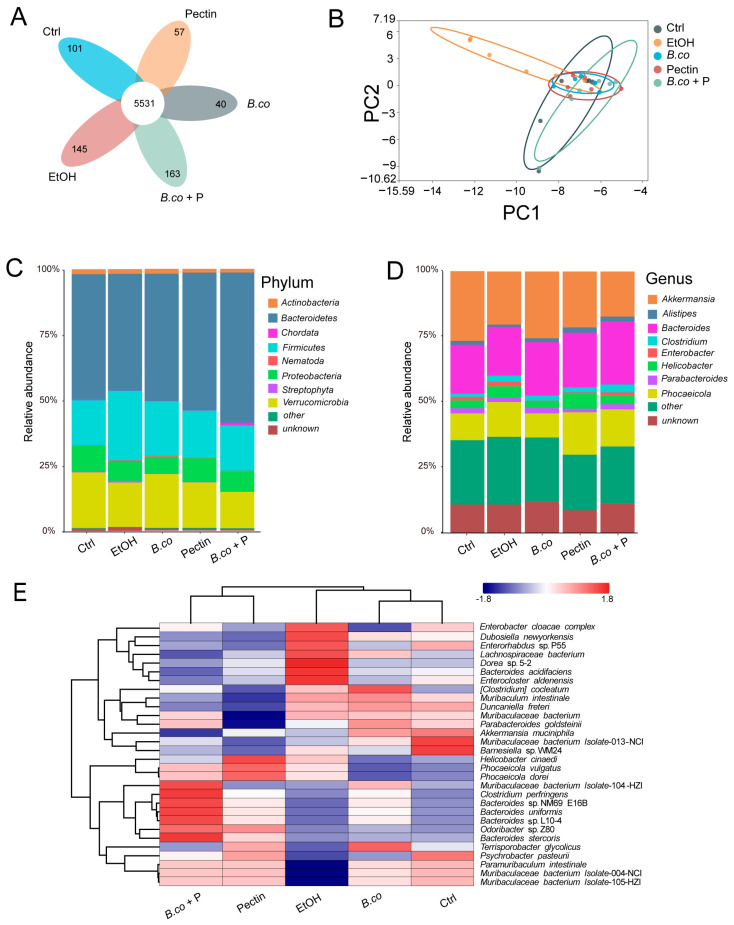
*B. coagulans*, pectin and the synbiotic regulated the composition of gut microbiota. (**A**) Venn diagram representing the common species between all five group. (**B**) Principal Components analysis of Bray–Curtis dissimilarities between all five group. (**C**) Fecal bacterial relative abundance at the phylum level. (**D**) The relative abundance of fecal bacterial in genus. (**E**) The heatmap of different species for *B. coagulans*, pectin and the synbiotic group. (n = 6 per group); Ctrl: healthy control group; EtOH: ethanol-induced group; *B. co*: supplementation of *B. coagulans* group; Pectin: supplementation of pectin group; *B. co* + P: supplementation of *B. coagulans* and pectin group.

Metagenomic sequencing of fecal samples assessed the impact of *B. coagulans*, pectin, and their synbiotic on the gut microbiota structure in ethanol-treated mice. Shared species analysis identified 5531 common species across the five groups (Figure 5A). Principal Components analysis (PCA) based on the Bray–Curtis distance showed revealed distinct clustering separating the healthy control group and ethanol-induced group (Figure 5B). Treatment groups (*B.coagulans*, pectin, synbiotic) also exhibited significant structural divergence from the ethanol-induced group, confirming their substantial impact. Furthermore, their community clustering showed greater similarity to the healthy group compared to the ethanol-induced group (Figure 5B). While phylum-level compositions was comparable across all groups, relative abundances varied significantly. The dominant phyla included *Actinobacteria*, *Bacteroidetes*, *Chordata*, *Firmicutes*, *Nematoda*, *Proteobacteria*, *Streptophyta* and *Verrucomicrobia* (Figure 5C). Ethanol exposure significantly decreased *Verrucomicrobia* and *Proteobacteria* abundance while increasing *Firmicutes* and *Streptophyta* compared to the healthy control group (Figure 5C,D), consistent with reported ALD dysbiosis [40,41]. Following *B. coagulans* treatment, *Verrucomicrobia* increased significantly while *Firmicutes* decreased significantly. Both pectin and synbiotic treatments led to a reduction in the abundance of *Firmicutes* and *Streptophyta* and an increase in *Verrucomicrobia* abundance compared to the ethanol group. At the genus level, dominant taxa included *Phocaeicola*, *Helicobacter*, *Bacteroides*, *Clostridium*, *Enterobacter*, and *Alistipes* (Figure 5D). Ethanol exposure significantly decreased beneficial bacterium *Akkermansia* and enriched the potential pathogenic bacterium *Helicobacter*. Furthermore, *B. coagulans* treatment restored the gut microbiota composition toward that of the healthy control group, demonstrating significant increases in beneficial bacterium *Akkermansia* abundance and decreases in potential pathogenic bacterium *Enterobacter* abundance relative to the ethanol-induced group. Pectin therapy resulted in significant improvements in the abundances of beneficial bacterium *Phocaeicola* and *Akkermansia*, along with a reduction in potential pathogenic bacterium *Enterobacter* levels. Compared to the ethanol-induced group, the synbiotic group showed an increase in *Bacteroides* abundance but no significant effect in *Akkermansia* abundance (Figure 5D). These results suggest that the three treatments may suppressed pathogenic bacteria and promoted beneficial microbial populations, thereby contributing to the gradual restoration of intestinal microbiota balance.

To characterize the distinct gut microbiota structures among ethanol-induced mice, three treatment groups, and the healthy control, we comparatively analyzed the intestinal microbiome across all five groups. Hierarchical clustering of species revealed distinct microbial taxa that varied in response to *B. coagulans*, pectin, and their synbiotic supplementation in ethanol-induced mice (Figure 5E). Specifically, the ethanol-treated group showed enrichment of *Dorea* sp., *Bacteroides acidifaciens*, *Enterocloster aldenensis* and *Lachnospiraceae bacterium*. In contrast, the *B. coagulans* treatment group exhibited increased abundance of *Terrisporobacter* and *Muribaculaceae*. The healthy control group had the highest levels of *Akkermansia*, *Muribaculaceae*, and *Barnesiella* sp. WM24. Additionally, both the pectin and synbiotic groups displayed a distinct enrichment pattern, predominantly featuring *Bacteroides* sp., *Odoribacter* sp., *Phocaeicola dorei*, and *Muribaculaceae*, which differentiated them from the other groups (Figure 5E). These results suggest that enrichment of beneficial bacteria in the *B. coagulans*, pectin, and their synbiotic treatment groups contributes to improved health outcomes. Notably, despite undetectable levels of *B. coagulans* in feces from the probiotic-administered group, attributable to its suboptimal intestinal colonization, the transient intestinal bioactivity of *B. coagulans* nonetheless confers therapeutic efficacy in mice ALD models.

**Figure 6:**
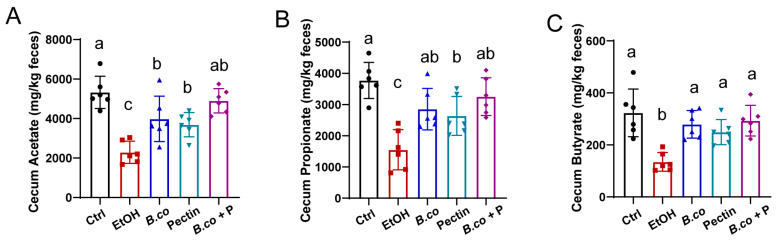
The SCFAs expression levels in fecal samples. Concentrations of fecal acetate (**A**), propionate (**B**), and butyrate (**C**). (n = 6 per group). Data are presented as means ± SEM. Values with distinct superscript letters (a, b and c) indicate significant differences (*p* < 0.05) as determined by one-way ANOVA followed by Tukey’s test. Ctrl: healthy control group; EtOH: ethanol-induced group; *B.co*: supplementation of *B. coagulans* group; Pectin: supplementation of pectin group; *B.co* + P: supplementation of *B. coagulans* and pectin group.

To further investigate effects on short-chain fatty acids (SCFAs) production, we quantified fecal acetate, propionate, and butyrate. Ethanol administration significantly reduced all three SCFAs, whereas *B. coagulans*, pectin, and their synbiotic treatments markedly increased SCFAs concentration compared to the ethanol group (Figure 6), indicating their association with enhanced gut SCFAs levels.

## 4. Discussion

Probiotic and prebiotic supplements that modulate gut microbiota, colonic epithelial integrity, and cytokine profiles are being actively investigated for their potential in preventing or treating ALD [42]. Intestinal dysbiosis and impaired barrier function are well-established drivers of inflammatory liver injury, and these phenomena are exacerbated in chronic alcohol abuse [1,43]. While extensive research has explored the gut microbiota’s role in maintaining host homeostasis and its potential therapeutic applications in ALD [14,43], our study specifically investigates how *B. coagulans*, pectin, and their synbiotic ameliorate ethanol-induced ALD in mice. Our findings demonstrate that the addition of *B. coagulans*, pectin and their synbiotic markedly alleviate ALD severity, as evidenced by restored intestinal barrier function, including upregulated tight junction proteins and reduced serum LPS, and improved hepatic pathology. The precise mechanism, we speculate this barrier restoration may involve SCFA-mediated signaling, given that SCFAs (elevated in treated groups) can activate GPR43/41 receptors on intestinal epithelial cells to enhance tight junction assembly [44,45]. This mechanism is supported by studies showing that *Akkermansia muciniphila* administration increases cecal acetate and propionate levels, which in turn activate GPR41/43 to promote intestinal stem cell proliferation and epithelial regeneration [46]. The reversal of ethanol-induced downregulation of TJs and Muc2 proteins, approaching levels in healthy group, further supports a synbiotic-driven repair mechanism, though the precise regulatory pathways (e.g., NF-κB or MAPK inhibition) require further validation.

In terms of inflammation, ethanol-induced LPS translocation activates Kupffer cells (KCs) via Toll-like receptor 4 (TLR4), triggering proinflammatory cytokines production (IL-1, TNF-α) that drives hepatocyte dysfunction and programmed cell death (PCD) [47,48]. Our observation that synbiotic treatment reduces these cytokines aligns with *B. coagulans*-mediated immunomodulation reported in prior studies, specifically, *B. coagulans* Unique IS-2 has been shown to decrease TNF-α and IL-1β levels in inflammatory bowel disease patients, likely through immunomodulatory pathways [26,27,49]. This anti-inflammatory effect may involve suppression of TLR4/NF-κB signaling, an avenue worthy of targeted mechanistic studies.

Consuming alcohol has been associated with alterations to the gut microbiota in humans, and dysbiosis is thought to be an essential variable in the development of ALD [20,50,51]. Regarding gut microbiota, ethanol-induced reduced of *A. muciniphila* (a key mucin-degrading bacterium) and enrichment of pathogenic bacteria (*E. aldenensis*) were reversed by our treatments (*B. coagulans* and/or pectin supplementation). The concurrent increase in SCFA-producing *Akkermansia* and *Muribaculaceae* suggests metabolic cross-feeding: *B. coagulans*, pectin, and their synbiotic may exert regulatory effects on the gut microbiota, act as antagonists against pathogenic bacteria, and facilitate intestinal barrier restoration [23,52]. This synergistic modulation aligns with evidence that *A. muciniphila* enhances mucus layer thickness and fosters beneficial microbial interactions [46], thereby contributing to barrier repair and anti-inflammatory effects.

While our study confirmed that *B. coagulans* combined with pectin exerts therapeutic effects on ALD in mice, several limitations remain. First, both the overall experimental duration and the supplementation period of *B. coagulans* were relatively short, which restricts conclusions regarding its long-term efficacy and colonization dynamics. The undetectable levels of *B. coagulans* in fecal samples further indicate its suboptimal intestinal retention, and the potential changes in its biological activity and therapeutic effects over an extended period remain unclear. Despite the observed therapeutic effects of the synbiotic, the inability to detect *B. coagulans* in feces raises questions about the exact mechanism by which it exerts its effects. It is possible that the bacterium exerts transient effects in the gut or influences the microbiota through metabolic product, but these hypotheses require further verification. Second, the lack of direct comparisons with other probiotic strains (e.g., *Lactobacillus*) or prebiotics (e.g., inulin) hinders the assessment of this synbiotic’s specificity, such as, co-administration of *Lactobacillus rhamnosus* with inulin has shown superior anti-inflammatory effects compared to single-agent treatments in patients with coronary artery disease [53], highlighting the necessity of comparative studies. Finally, the mechanistic links between gut microbiota alterations and molecular targets (e.g., TLR4, GPR43) remain unclear and require further validation using germ-free or gene-knockout models to identify specific targets. These issues should be prioritized in future research.

The interplay between probiotics, prebiotics, and ALD has gained substantial research attention, and our study provides empirical evidence for this connection in a mice model. Specifically, we induced ALD in mice via ethanol administration and evaluated the therapeutic effects of *B. coagulans*, pectin, and their synbiotic. Our findings demonstrate that these interventions: (i) Modulate gut microbiota structure: they enhanced microbial richness and diversity, with significant enrichment of SCFA-producing taxa (e.g., *Terrisporobacter*, *Muribaculaceae*, *Akkermansia*, and *Phocaeicola dorei*) in treated groups, suggesting direct or indirect regulation of ALD through microbiota-mediated pathways [54]. (ii) Strengthen intestinal barrier function: by upregulating intestinal mucus and intestinal epithelial tight junction proteins, the interventions mitigated ethanol-induced intestinal leakage, a key driver of liver inflammation [26,31,33]. (iii) Promote metabolic and immune homeostasis: elevated SCFA production and reduced proinflammatory cytokines collectively contributed to liver protection. Notably, the synbiotic (*B. coagulans* + pectin) exerted superior effects compared to single treatments, confirming our hypothesis that their combination alleviates ethanol-induced liver injury by restoring intestinal barrier integrity, modulating gut microbiota, boosting SCFAs production, and regulating cytokines profiles. These findings support the synbiotic’s potential in ALD by targeting microbiota-liver crosstalk, but future work addressing these limitations will strengthen clinical translatability.

## Data Availability

The original contributions presented in this study are included in the article/Supplementary Material. Further inquiries can be directed to the corresponding author.

## Supplementary Materials

The following supporting information can be downloaded at: https://www.mdpi.com/article/10.3390/microorganisms13091986/s1, Table S1: Primers used for RT-qPCR.

## References

[1] Wu X., Fan X., Miyata T., Kim A., Cajigas-Du Ross C.K., Ray S., Huang E., Taiwo M., Arya R., Wu J. (2023). Recent Advances in Understanding of Pathogenesis of Alcohol-Associated Liver Disease. Annu. Rev. Pathol..

[2] WHO (2024). Global Status Report on Alcohol and Health 2024.

[3] Seitz H.K., Bataller R., Cortez-Pinto H., Gao B., Gual A., Lackner C., Mathurin P., Mueller S., Szabo G., Tsukamoto H. (2018). Alcoholic liver disease. Nat. Rev. Dis. Primers.

[4] Patel R., Mueller M. (2025). StatPearls.

[5] Ceni E., Mello T., Galli A. (2014). Pathogenesis of alcoholic liver disease: Role of oxidative metabolism. World J. Gastroenterol..

[6] Seitz H.K. (2020). The role of cytochrome P4502E1 in the pathogenesis of alcoholic liver disease and carcinogenesis. Chem. Biol. Interact..

[7] Gao B., Ahmad M.F., Nagy L.E., Tsukamoto H. (2019). Inflammatory pathways in alcoholic steatohepatitis. J. Hepatol..

[8] Coe C., Patel A., Lawrence D. (2023). Pharmacotherapy options for alcohol use disorder in patients with alcohol-associated liver disease: A brief guide for clinicians. Clin. Liver Dis..

[9] Hill C., Guarner F., Reid G., Gibson G.R., Merenstein D.J., Pot B., Morelli L., Canani R.B., Flint H.J., Salminen S. (2014). The International Scientific Association for Probiotics and Prebiotics consensus statement on the scope and appropriate use of the term probiotic. Nat. Rev. Gastroenterol. Hepatol..

[10] Sanders M.E., Merenstein D.J., Reid G., Gibson G.R., Rastall R.A. (2019). Probiotics and prebiotics in intestinal health and disease: From biology to the clinic. Nat. Rev. Gastroenterol. Hepatol..

[11] Cameron D., Hock Q.S., Kadim M., Mohan N., Ryoo E., Sandhu B., Yamashiro Y., Jie C., Hoekstra H., Guarino A. (2017). Probiotics for gastrointestinal disorders: Proposed recommendations for children of the Asia-Pacific region. World J. Gastroenterol..

[12] King S., Glanville J., Sanders M.E., Fitzgerald A., Varley D. (2014). Effectiveness of probiotics on the duration of illness in healthy children and adults who develop common acute respiratory infectious conditions: A systematic review and meta-analysis. Br. J. Nutr..

[13] Szymański H., Szajewska H. (2019). Lack of Efficacy of Lactobacillus reuteri DSM 17938 for the Treatment of Acute Gastroenteritis: A Randomized Controlled Trial. Pediatr. Infect. Dis. J..

[14] Gu Z., Liu Y., Hu S., You Y., Wen J., Li W., Wang Y. (2019). Probiotics for Alleviating Alcoholic Liver Injury. Gastroenterol. Res. Pract..

[15] Leclercq S., Stärkel P., Delzenne N.M., de Timary P. (2019). The gut microbiota: A new target in the management of alcohol dependence?. Alcohol.

[16] Gibson G.R., Hutkins R., Sanders M.E., Prescott S.L., Reimer R.A., Salminen S.J., Scott K., Stanton C., Swanson K.S., Cani P.D. (2017). Expert consensus document: The International Scientific Association for Probiotics and Prebiotics (ISAPP) consensus statement on the definition and scope of prebiotics. Nat. Rev. Gastroenterol. Hepatol..

[17] Vernon J.J. (2025). Modulation of the Human Microbiome: Probiotics, Prebiotics, and Microbial Transplants. Adv. Exp. Med. Biol..

[18] Shahramian I., Kalvandi G., Javaherizadeh H., Khalili M., Noori N.M., Delaramnasab M., Bazi A. (2018). The effects of prebiotic supplementation on weight gain, diarrhoea, constipation, fever and respiratory tract infections in the first year of life. J. Paediatr. Child Health.

[19] Boehm G., Jelinek J., Stahl B., van Laere K., Knol J., Fanaro S., Moro G., Vigi V. (2004). Prebiotics in infant formulas. J. Clin. Gastroenterol..

[20] Grander C., Adolph T.E., Wieser V., Lowe P., Wrzosek L., Gyongyosi B., Ward D.V., Grabherr F., Gerner R.R., Pfister A. (2018). Recovery of ethanol-induced *Akkermansia muciniphila* depletion ameliorates alcoholic liver disease. Gut.

[21] Shukla P.K., Meena A.S., Manda B., Gomes-Solecki M., Dietrich P., Dragatsis I., Rao R. (2018). Lactobacillus plantarum prevents and mitigates alcohol-induced disruption of colonic epithelial tight junctions, endotoxemia, and liver damage by an EGF receptor-dependent mechanism. FASEB J..

[22] Jurenka J.S. (2012). *Bacillus coagulans*: Monograph. Altern. Med. Rev. J. Clin. Ther..

[23] Mu Y., Cong Y. (2019). *Bacillus coagulans* and its applications in medicine. Benef. Microbes.

[24] Zhao Y., Dong B.R., Hao Q. (2022). Probiotics for preventing acute upper respiratory tract infections. Cochrane Database Syst. Rev..

[25] Shinde T., Vemuri R., Shastri M.D., Perera A.P., Tristram S., Stanley R., Eri R. (2019). Probiotic *Bacillus coagulans* MTCC 5856 spores exhibit excellent in-vitro functional efficacy in simulated gastric survival, mucosal adhesion and immunomodulation. J. Funct. Foods.

[26] Liu Z., Jiang Z., Zhang Z., Liu T., Fan Y., Liu T., Peng N. (2022). *Bacillus coagulans* in Combination with Chitooligosaccharides Regulates Gut Microbiota and Ameliorates the DSS-Induced Colitis in Mice. Microbiol. Spectr..

[27] Shinde T., Perera A.P., Vemuri R., Gondalia S.V., Beale D.J., Karpe A.V., Shastri S., Basheer W., Southam B., Eri R. (2020). Synbiotic supplementation with prebiotic green banana resistant starch and probiotic *Bacillus coagulans* spores ameliorates gut inflammation in mouse model of inflammatory bowel diseases. Eur. J. Nutr..

[28] Liu Z., Liu T., Zhang Z., Fan Y. (2024). *Bacillus coagulans* regulates gut microbiota and ameliorates the alcoholic-associated liver disease in mice. Front. Microbiol..

[29] Yan A.W., Fouts D.E., Brandl J., Stärkel P., Torralba M., Schott E., Tsukamoto H., Nelson K.E., Brenner D.A., Schnabl B. (2011). Enteric dysbiosis associated with a mouse model of alcoholic liver disease. Hepatology.

[30] Ferrere G., Wrzosek L., Cailleux F., Turpin W., Puchois V., Spatz M., Ciocan D., Rainteau D., Humbert L., Hugot C. (2017). Fecal microbiota manipulation prevents dysbiosis and alcohol-induced liver injury in mice. J. Hepatol..

[31] Wrzosek L., Ciocan D., Hugot C., Spatz M., Dupeux M., Houron C., Lievin-Le Moal V., Puchois V., Ferrere G., Trainel N. (2021). Microbiota tryptophan metabolism induces aryl hydrocarbon receptor activation and improves alcohol-induced liver injury. Gut.

[32] Tian L., Scholte J., Borewicz K., van den Bogert B., Smidt H., Scheurink A.J., Gruppen H., Schols H.A. (2016). Effects of pectin supplementation on the fermentation patterns of different structural carbohydrates in rats. Mol. Nutr. Food Res..

[33] Hu W., Cassard A.M., Ciocan D. (2022). Pectin in Metabolic Liver Disease. Nutrients.

[34] Chiu W.C., Huang Y.L., Chen Y.L., Peng H.C., Liao W.H., Chuang H.L., Chen J.R., Yang S.C. (2015). Synbiotics reduce ethanol-induced hepatic steatosis and inflammation by improving intestinal permeability and microbiota in rats. Food Funct..

[35] Bertola A., Mathews S., Ki S.H., Wang H., Gao B. (2013). Mouse model of chronic and binge ethanol feeding (the NIAAA model). Nat. Protoc..

[36] Wang L., Fouts D.E., Stärkel P., Hartmann P., Chen P., Llorente C., DePew J., Moncera K., Ho S.B., Brenner D.A. (2016). Intestinal REG3 Lectins Protect against Alcoholic Steatohepatitis by Reducing Mucosa-Associated Microbiota and Preventing Bacterial Translocation. Cell Host Microbe.

[37] Zhao G., Nyman M., Jönsson J.A. (2006). Rapid determination of short-chain fatty acids in colonic contents and faeces of humans and rats by acidified water-extraction and direct-injection gas chromatography. Biomed. Chromatogr..

[38] Chen P., Torralba M., Tan J., Embree M., Zengler K., Stärkel P., van Pijkeren J.P., DePew J., Loomba R., Ho S.B. (2015). Supplementation of saturated long-chain fatty acids maintains intestinal eubiosis and reduces ethanol-induced liver injury in mice. Gastroenterology.

[39] Szabo G., Bala S., Petrasek J., Gattu A. (2010). Gut-liver axis and sensing microbes. Dig. Dis..

[40] Mutlu E.A., Gillevet P.M., Rangwala H., Sikaroodi M., Naqvi A., Engen P.A., Kwasny M., Lau C.K., Keshavarzian A. (2012). Colonic microbiome is altered in alcoholism. Am. J. Physiol. Gastrointest. Liver Physiol..

[41] Llopis M., Cassard A.M., Wrzosek L., Boschat L., Bruneau A., Ferrere G., Puchois V., Martin J.C., Lepage P., Le Roy T. (2016). Intestinal microbiota contributes to individual susceptibility to alcoholic liver disease. Gut.

[42] Patel D., Desai C., Singh D., Soppina V., Parwani K., Patel F., Mandal P. (2022). Synbiotic Intervention Ameliorates Oxidative Stress and Gut Permeability in an In Vitro and In Vivo Model of Ethanol-Induced Intestinal Dysbiosis. Biomedicines.

[43] Dukić M., Radonjić T., Jovanović I., Zdravković M., Todorović Z., Kraišnik N., Aranđelović B., Mandić O., Popadić V., Nikolić N. (2023). Alcohol, Inflammation, and Microbiota in Alcoholic Liver Disease. Int. J. Mol. Sci..

[44] Kim M., Friesen L., Park J., Kim H.M., Kim C.H. (2018). Microbial metabolites, short-chain fatty acids, restrain tissue bacterial load, chronic inflammation, and associated cancer in the colon of mice. Eur. J. Immunol..

[45] Visekruna A., Luu M. (2021). The Role of Short-Chain Fatty Acids and Bile Acids in Intestinal and Liver Function, Inflammation, and Carcinogenesis. Front. Cell Dev. Biol..

[46] Kim S., Shin Y.C., Kim T.Y., Kim Y., Lee Y.S., Lee S.H., Kim M.N., O E., Kim K.S., Kweon M.N. (2021). Mucin degrader *Akkermansia muciniphila* accelerates intestinal stem cell-mediated epithelial development. Gut Microbes.

[47] Nagy L.E., Ding W.X., Cresci G., Saikia P., Shah V.H. (2016). Linking Pathogenic Mechanisms of Alcoholic Liver Disease with Clinical Phenotypes. Gastroenterology.

[48] Wang H., Mehal W., Nagy L.E., Rotman Y. (2021). Immunological mechanisms and therapeutic targets of fatty liver diseases. Cell. Mol. Immunol..

[49] Shinde T., Perera A.P., Vemuri R., Gondalia S.V., Karpe A.V., Beale D.J., Shastri S., Southam B., Eri R., Stanley R. (2019). Synbiotic Supplementation Containing Whole Plant Sugar Cane Fibre and Probiotic Spores Potentiates Protective Synergistic Effects in Mouse Model of IBD. Nutrients.

[50] Hsu C.L., Schnabl B. (2023). The gut-liver axis and gut microbiota in health and liver disease. Nat. Rev. Microbiol..

[51] Macnaughtan J., Jalan R. (2015). Clinical and pathophysiological consequences of alterations in the microbiome in cirrhosis. Am. J. Gastroenterol..

[52] Abdhul K., Ganesh M., Shanmughapriya S., Vanithamani S., Kanagavel M., Anbarasu K., Natarajaseenivasan K. (2015). Bacteriocinogenic potential of a probiotic strain *Bacillus coagulans* [BDU3] from Ngari. Int. J. Biol. Macromol..

[53] Moludi J., Khedmatgozar H., Nachvak S.M., Abdollahzad H., Moradinazar M., Sadeghpour Tabaei A. (2022). The effects of co-administration of probiotics and prebiotics on chronic inflammation, and depression symptoms in patients with coronary artery diseases: A randomized clinical trial. Nutr. Neurosci..

[54] Martin-Gallausiaux C., Marinelli L., Blottiere H.M., Larraufie P., Lapaque N. (2021). SCFA: Mechanisms and functional importance in the gut. Proc. Nutr. Soc..

